# Editorial: Nanodrug delivery strategies for enhanced cancer chemo-immunotherapy

**DOI:** 10.3389/fbioe.2025.1653425

**Published:** 2025-07-08

**Authors:** Yufen Xiao

**Affiliations:** ^1^ Department of Biomedical Engineering, Simmons Comprehensive Cancer Center, Program in Genetic Drug Engineering, University of Texas Southwestern Medical Center, Dallas, TX, United States; ^2^ Department of Biochemistry, Simmons Comprehensive Cancer Center, Program in Genetic Drug Engineering, University of Texas Southwestern Medical Center, Dallas, TX, United States

**Keywords:** drug delivery, nanotechnology, pharmaceutics, cancer, immunotherapy

The rapid development in nanotechnology has led to a revolution in nanomedicine, particularly in the field of drug delivery for cancer treatment. Engineering biomaterials with new features enables precision and effective therapies compared to conventional therapies. This invited Research Topic contains 5 articles, including 2 original research papers, 2 review articles, and 1 minireview article, contributed by 36 researchers worldwide, which covers a wide-range of Research Topics in the fields of nanomedicine and cancer treatment, including peptide based vesicles (Yu et al.), nanoliposomes (Saberian et al.), lipid nanoparticles (Zhao et al.), and others (Zhou et al.).

Cancer therapies have been evolving with advances in oncology. This invited Research Topic encompasses both reviews and original research articles, ranging from immunotherapy to synergistic therapy. Nanomedicine and nanotherapy represent cutting-edge research in treatment, offering prolonged circulation, reduced off-target effects, and enhanced therapeutic efficacy, making significant strides in oncological care. Among them, active targeting strategies were employed to enhance the anti-cancer efficacy. For instance, a folate receptor (FR)-mediated approach was developed by Saberian et al. that offered a selective strategy to target cancer cells. The folate-targeted Bleomycin nanoliposomes successfully enhanced the cellular uptake, thereby improving antitumor activity against oral cancer cells. Cancer cells often overexpress glucose transport protein 1 (GLUT1), facilitating increased glucose uptake and fueling their rapid growth and proliferation. In this backdrop, Zhao et al. incorporated ginsenoside RG3 (RG3), a GLUT1 substrate that possessed a hydrophilic sugar moiety and hydrophobic steroidal structure, into lipid nanoparticles to specifically target the overexpressed GLUT1 on the malignant fibroblast membrane.

Cancer immunotherapy represents a significant advancement with growing attention owing to the satisfactory results in hematologic tumors, melanoma, and other tumors. The immune checkpoint blockade therapy, cytokine therapy, and chimeric antigen receptor T cells have shown exciting clinical outcomes. However, the strong immunosuppressive microenvironment of highly malignant tumors, e.g., osteosarcoma, greatly hinders the therapeutic efficacy of immunotherapy. Thereby, Lian et al. review the role of immune cells in the progression of osteosarcoma and describe regulatory strategies tailored to the characteristics of different immune cell types that can enhance the efficacy of immunotherapy. Activating the local and systemic immune system by delivering tumor-associated antigens and tumor-specific cytokines offers opportunities for immunotherapy. Combination of immunotherapy with other cancer therapies, such as chemotherapy, chemodynamic therapy, and photothermal therapy, represents one of the current research breakthroughs. On the other hand, Yu et al. summarized the design of peptide based vesicle for cancer immunotherapy from a viewpoint of material science. The combination of peptides and vesicles integrates the innate immunological functions of cellular vesicles and antigen and immune modulator features of peptides, enhancing the efficacy of the immune response and anti-cancer effect. In another review article, Zhou et al. refined the advanced nanotechnology in the treatment of head and neck cancer, providing insights into precision therapy mediated by advanced nanomaterials.

Overall, the current Research Topic reports a diverse intersection of nanotechnologies that have demonstrated promising results in engineering nanomaterials and cancer microenvironments for cancer therapy. This interdisciplinary research effectively bridges fundamental materials science development and clinical medical translation, offering significant benefits to the fields of nanotechnology, biomedical engineering, and pharmaceutics.

